# Establishment of Induced Pluripotent Stem Cells from Centenarians for Neurodegenerative Disease Research

**DOI:** 10.1371/journal.pone.0041572

**Published:** 2012-07-25

**Authors:** Takuya Yagi, Arifumi Kosakai, Daisuke Ito, Yohei Okada, Wado Akamatsu, Yoshihiro Nihei, Akira Nabetani, Fuyuki Ishikawa, Yasumichi Arai, Nobuyoshi Hirose, Hideyuki Okano, Norihiro Suzuki

**Affiliations:** 1 Department of Neurology, School of Medicine, Keio University, Tokyo, Japan; 2 Department of Physiology, School of Medicine, Keio University, Tokyo, Japan; 3 Kanrinmaru Project, School of Medicine, Keio University, Tokyo, Japan; 4 Laboratory of Cell Cycle Regulation, Department of Gene Mechanisms, Graduate School of Biostudies, Kyoto University, Kyoto, Japan; 5 Division of Geriatric Medicine, Department of Internal Medicine, School of Medicine, Keio University, Tokyo, Japan; Kyushu University, Japan

## Abstract

Induced pluripotent stem cell (iPSC) technology can be used to model human disorders, create cell-based models of human diseases, including neurodegenerative diseases, and in establishing therapeutic strategies. To detect subtle cellular abnormalities associated with common late-onset disease in iPSCs, valid control iPSCs derived from healthy donors free of serious late-onset diseases are necessary. Here, we report the generation of iPSCs from fibroblasts obtained immediately postmortem from centenarian donors (106- and 109-years-old) who were extremely healthy until an advanced age. The iPSCs were generated using a conventional method involving OCT4, SOX2, KLF4, and c-MYC, and then differentiated into neuronal cells using a neurosphere method. The expression of molecules that play critical roles in late-onset neurodegenerative diseases by neurons differentiated from the centenarian-iPSCs was compared to that of neurons differentiated from iPSCs derived from familial Alzheimer's disease and familial Parkinson's disease (PARK4: triplication of the α synuclein gene) patients. The results indicated that our series of iPSCs would be useful in neurodegeneration research. The iPSCs we describe, which were derived from donors with exceptional longevity who were presumed to have no serious disease risk factors, would be useful in longevity research and as valid super-controls for use in studies of various late-onset diseases.

## Introduction

In 2006, Takahashi et al. directly reprogrammed somatic cells into induced pluripotent stem cells (iPSCs), thereby opening a novel approach to disease modeling and drug discovery [1], [2]. The availability of iPSCs is particularly advantageous for research involving neurological diseases, since it is difficult to obtain affected tissues from living patients. A new era in *in vitro* modeling of neurodegenerative diseases recently began when iPSC technology was used to recapitulate the phenotypes of several late-onset neurodegenerative diseases, such as Alzheimer's disease (AD) and Parkinson's disease (PD), diseases in which clinical signs appear on or after presenium [3]–[8]. However, a major obstacle to iPSC studies of common late-onset neurodegenerative diseases remains the lack of appropriate control iPSCs. Because most studies conducted to date using patient-specific iPSCs have focused on congenital or hereditary diseases, the control iPSCs used, which are derived from so-called healthy volunteers, are not excluded from the risks associated with common late-onset diseases. To detect subtle biochemical and/or cellular abnormalities associated with common late-onset diseases using iPSCs, super-control iPSCs derived from extremely healthy donors clinically determined to be free of serious late-onset diseases are necessary. Very elderly people who are in excellent health would be expected to present few risk factors for late-onset neurodegenerative diseases, and would thus represent a valid control population [9]. Lapasset et al. recently generated iPSCs derived from senescent cells and cells from a centenarian (101-year-old) individual by introducing six factors: OCT4, SOX2, KLF4, c-MYC, NANOG, and LIN28 [10]. The reprogramming strategy used in that study was designed to overcome cellular senescence, which represents a critical barrier to reprogramming. Unfortunately, no clinical information is available indicating whether these iPSCs would be suitable for use as a control for late-onset diseases.

Here, we report the establishment of iPSCs using cells derived from centenarians who were in exceptionally good health until an advanced age. The iPSCs were established using a conventional method involving the introduction of four factors: OCT4, SOX2, KLF4, and c-MYC, and were differentiated into neuronal cells using the neurosphere method [11]. The expression of several molecules critical to the development of late-onset neurodegenerative diseases, such as β-amyloid (Aβ), α-synuclein, and tau, was compared between neurons differentiated from our centenarian-derived iPSCs and neurons differentiated from iPSCs derived from familial AD (FAD) and PARK4 patients. Our results indicate that iPSCs derived from centenarians represent a valid super-control for use in studies of late-onset diseases, and that these cells could also be useful in longevity research.

## Results

### Generation of iPSCs derived from a donor with exceptional longevity

We established two iPSC clones (100–1 #8 and #16) derived from a 106-year-old donor and one clone (100–2 #1) derived from a 109-year-old donor using retroviral transduction of primary human fibroblasts with the original Yamanaka factors OCT4, SOX2, KLF4, and c-MYC. Dermal fibroblasts were obtained postmortem using protocols approved by Keio University after informed consent was obtained from the families of the donors. Based on medical history and detailed interviews with family members, a neurologist determined that the donors had not suffered from dementia, movement disorders, or other serious diseases until 100 years old (Text S1). Although a recent study demonstrated that reprogramming of senescent fibroblasts using the four gene combination of OCT4, SOX2, NANOG, and LIN28 fails to generate iPSCs, we were able to easily generate three iPSC clones from the centenarian donors using these four factors, suggesting that the original Yamanaka factors are sufficient for reprogramming cells from even extremely aged individuals [1].

**Figure 1:**
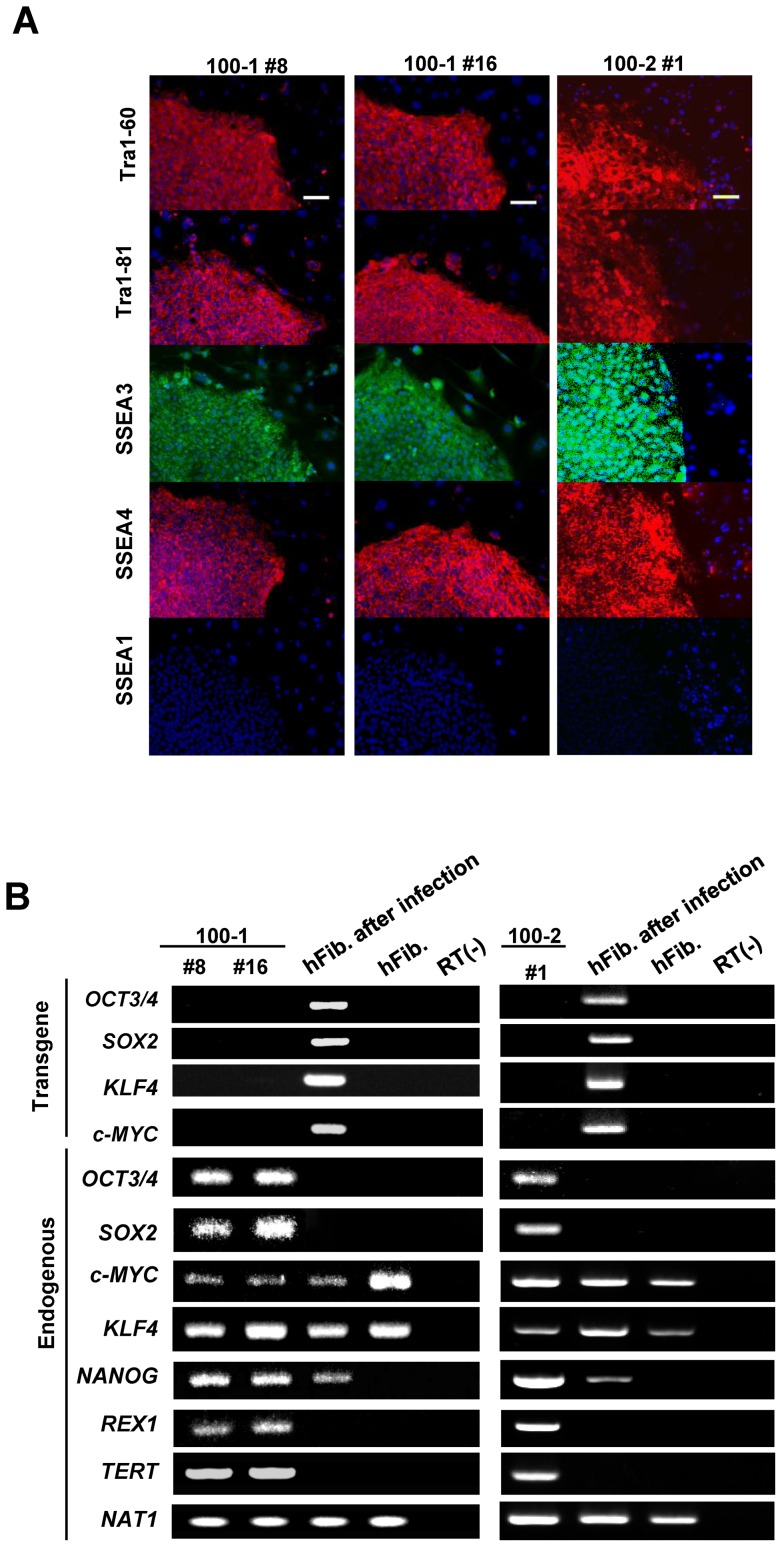
Generation of 100–1 and 100–2 iPSCs from centenarian donor fibroblasts. (A) Both the 100–1 and 100–2 iPSC lines exhibit markers of pluripotency. All iPSCs expressed the pluripotency markers Tra-1–60, Tra-1–81, SSEA3, and SSEA4. Nuclei were stained with DAPI. Bar  = 200 μm. (B) RT-PCR analysis of the transgenes OCT3/4, SOX2, KLF4, c-MYC and the endogenous hESC marker genes. Donor fibroblasts examined 6 days after transduction with the retroviruses are positive for the transgenes. RNA was extracted from the 100–1 and 100–2 iPSCs at passage 10.

**Figure 2:**
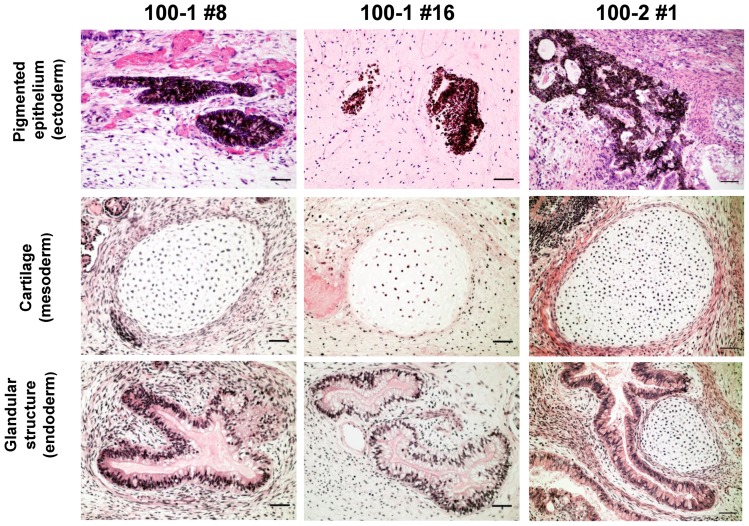
Teratomas derived from SCID mice injected with 100–1 and 100–2 iPSCs. Gross morphology and hematoxylin and eosin staining of representative teratomas generated from iPSCs 100–1 #8, 100–1 #16, and 100–2 #1. Tissues representing all 3 embryonic germ layers, including pigmented epithelium (ectoderm), cartilage (mesoderm), and glandular structure (endoderm) are shown. Bar  = 50 μm.

All the centenarian-iPSC clones demonstrated typical characteristics of pluripotent stem cells: similar morphology to embryonic stem cells (ESCs), expression of pluripotency markers, including Tra-1–60, Tra-1–81, SSEA3, and SSEA4 (Fig. 1A), silencing of retroviral transgenes, and reactivation of genes indicative of pluripotency (Fig. 1B). The capacity of the centenarian-iPSC clones to differentiate was confirmed *in vivo* by teratoma formation and *in vitro* by differentiation via embryoid bodies (Fig. 2 and Figure S1). To validate our reprogramming technique, we conducted a comprehensive analysis of the 100–1 #16 iPSC clone. A heat map analysis showed that the global gene expression profile of this clone, including expression of genes closely associated with longevity (insulin-like growth factor 1 (igf1), IGF-like family receptor 1 (igflr1), sirtuin 1 and 2 (sirt 1 and 2), and forkhead box O1 (foxo1)), was similar to the gene expression profile of the well-established iPSC line 201B7 originally derived from a 36-year-old Caucasian female [1] (Figure S2). An array comparative genomic hybridization (aCGH) analysis of clones 100–1 #8 and 100–1 #16 showed that there were 308 and 274 copy number aberrations, respectively, out of about 17,000 locations (Table S1).

**Figure 3:**
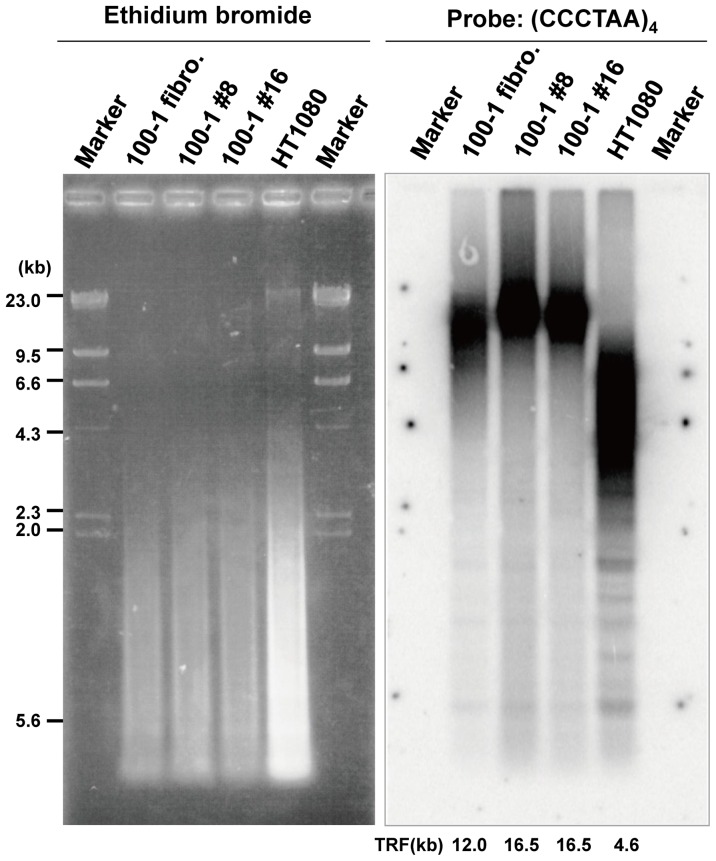
Analysis of centenarian-iPSC telomere length. Southern blot showing telomere length in 100–1 #8 (passage 10) and 100–1 #16 (passage 12) iPSCs compared to that of their parental fibroblast (passage 5). HT1080 served as a control. TRF lengths were calculated by comparing the electromobility of sample TRFs to that of makers. Values are displayed in kilobases (kb) below the figure.

Telomere shortening is thought to be linked to the aging process, but telomere length is known to increase in iPSCs after reprogramming as a result of telomerase activation. To confirm that telomere elongation had occurred, we measured the telomere length of iPSCs from clones 100–1 #8 and 100–1 #16 and found that the mean telomere lengths (both terminal restriction fragment (TRF) lengths were ∼16.5 kb) were greater than that of the parent fibroblasts (∼12.0 kb) (Fig. 3). In summary, using the reprogramming method originally described by Takahashi et al. [1], [2], we established iPSCs from dermal fibroblasts obtained postmortem from centenarian donors, indicating that extreme longevity of a donor does not preclude nuclear reprogramming.

### Differentiation of centenarian-derived iPSCs into neurons

**Figure 4:**
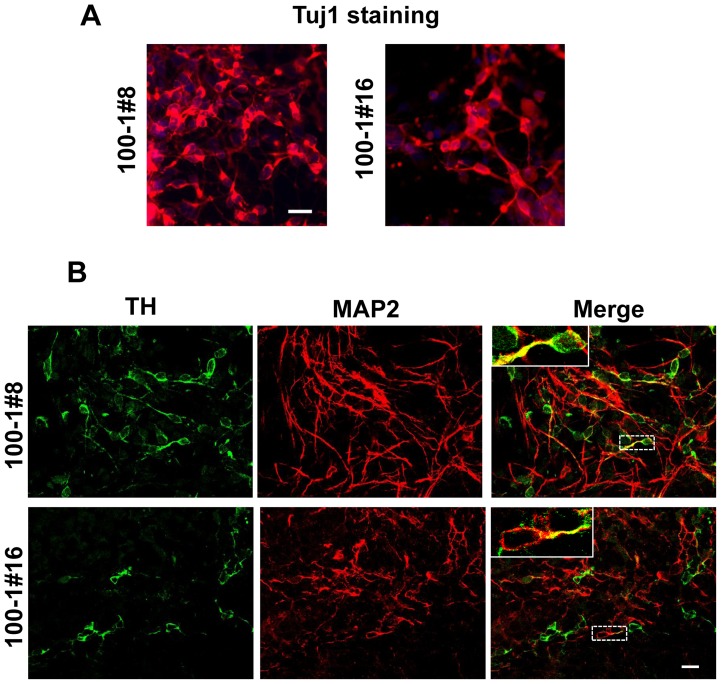
Differentiation of centenarian-iPSCs into neurons. (A) Neural differentiation of iPSCs 100–1 #8 and 100–1 #16. Representative images of immunocytochemical staining for the early neuronal marker βIII-tubulin following neural differentiation. (B) Confocal images of co-staining with the mature neuron marker MAP-2 and the dopaminergic and noradrenergic neuronal marker tyrosine hydroxylase (TH). Inserts show high magnifications of dotted white squares. Bar  = 20 μm. Cells were counterstained with DAPI (blue).

Differentiation of iPSCs derived from centenarian donors into neurons allows for the expansion of *in vitro* investigations of neurodegeneration through comparisons of disease-specific iPSCs. To determine whether the aging of donor cells affects neuronal differentiation, both the 100–1 #8 and 100–1 #16 iPSC lines were induced to differentiate into neural cells [6], [12], [13], and were cultured on Matrigel-coated dishes for 2 weeks to induce terminal differentiation. As shown in Figure 4A, about 80% of the neural cells differentiated from centenarian-derived iPSCs stained positive for βIII-tubulin, and we detected no obvious differences with our previous study with respect to generation of neurons from these cells [6]. Furthermore, the differentiated neurons were positive for the neuronal differentiation marker MAP2 and the dopaminergic and noradrenergic neuron marker tyrosine hydroxylase (TH) (Fig. 4B). These data indicated that iPSCs derived from centenarian donor cells differentiate into neurons *in vitro* in the same manner as other established iPSCs.

### Production of neurodegeneration-related molecules on neurons differentiated from centenarian-iPSCs

**Figure 5:**
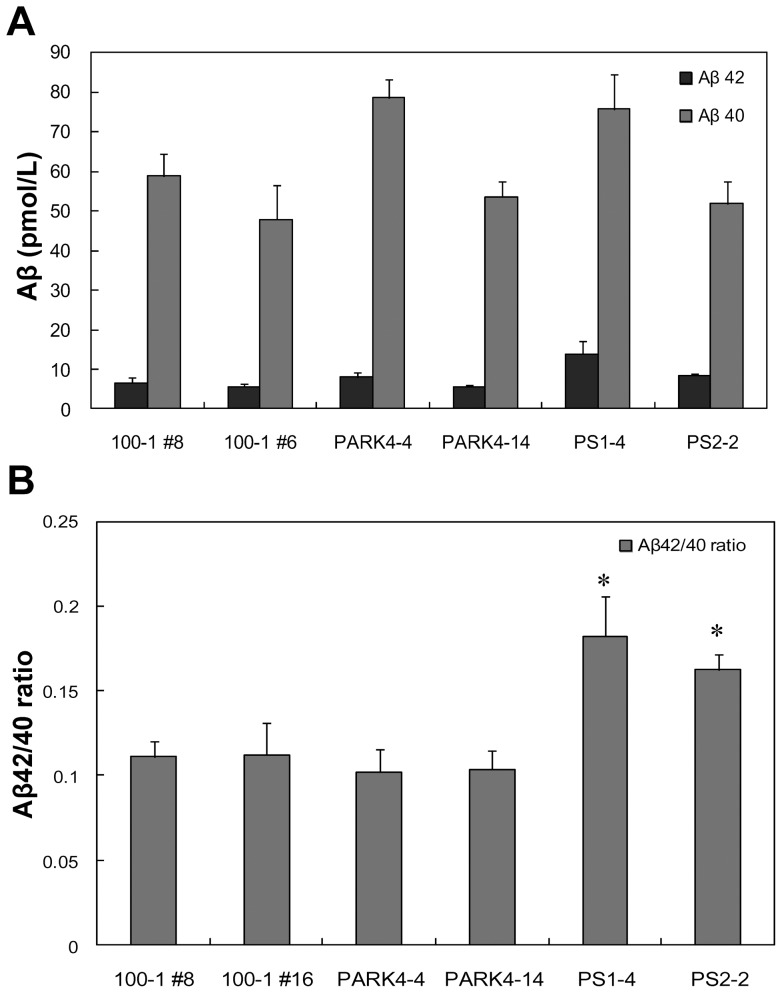
Characterization of the neurodegeneration-related molecules Aβ, α-synuclein, and tau protein in neurons differentiated from centenarian-iPSCs. (A) Secretion of Aβ40 and Aβ42 from neurons differentiated from iPSCs 100–1 #8 and 100–1 #16, and the neurodegenerative disease-specific iPSCs PARK4-4, PARK4-14, PS1-4, and PS2-2. (B) The ratio of Aβ42/Aβ40 secreted from neurons differentiated from 100–1 iPSCs and neurodegenerative disease-specific iPSCs. The ratio of Aβ42/Aβ40 secreted by neurons differentiated from both the PS1 and PS2 iPSCs was significantly higher than that of neurons differentiated from the other iPSCs. Significant differences among groups were examined using one-way analysis of variance followed by Tukey-Kramer's post hoc test (*P<0.05).

To characterize centenarian-iPSCs from a neuropathological standpoint, we compared the secretion of Aβ from neurons differentiated from 100–1 #8 and 100–1 #16 iPSCs to that of neurons differentiated from established AD- and PD-specific iPSCs. As shown Figure 5A, there was no significant difference between the various cell types with regard to the total levels of Aβ40 or Aβ42 in the medium. The ratio of Aβ42 to Aβ40 was identical for the neurons differentiated from the centenarian- and PARK4-iPSCs (PARK4-4, PARK4-14), whereas the Aβ42/Aβ40 ratio for the FAD-specific neurons (PS1-4, PS2-2) was significantly higher than for the other neurons (Fig. 5B). These data are consistent with our previous results [6], and indicate that the ratio of secreted Aβ42 to Aβ40 is disease-specific and thus may represent a potential diagnostic criterion for neurons derived from patient iPSCs.

**Figure 6:**
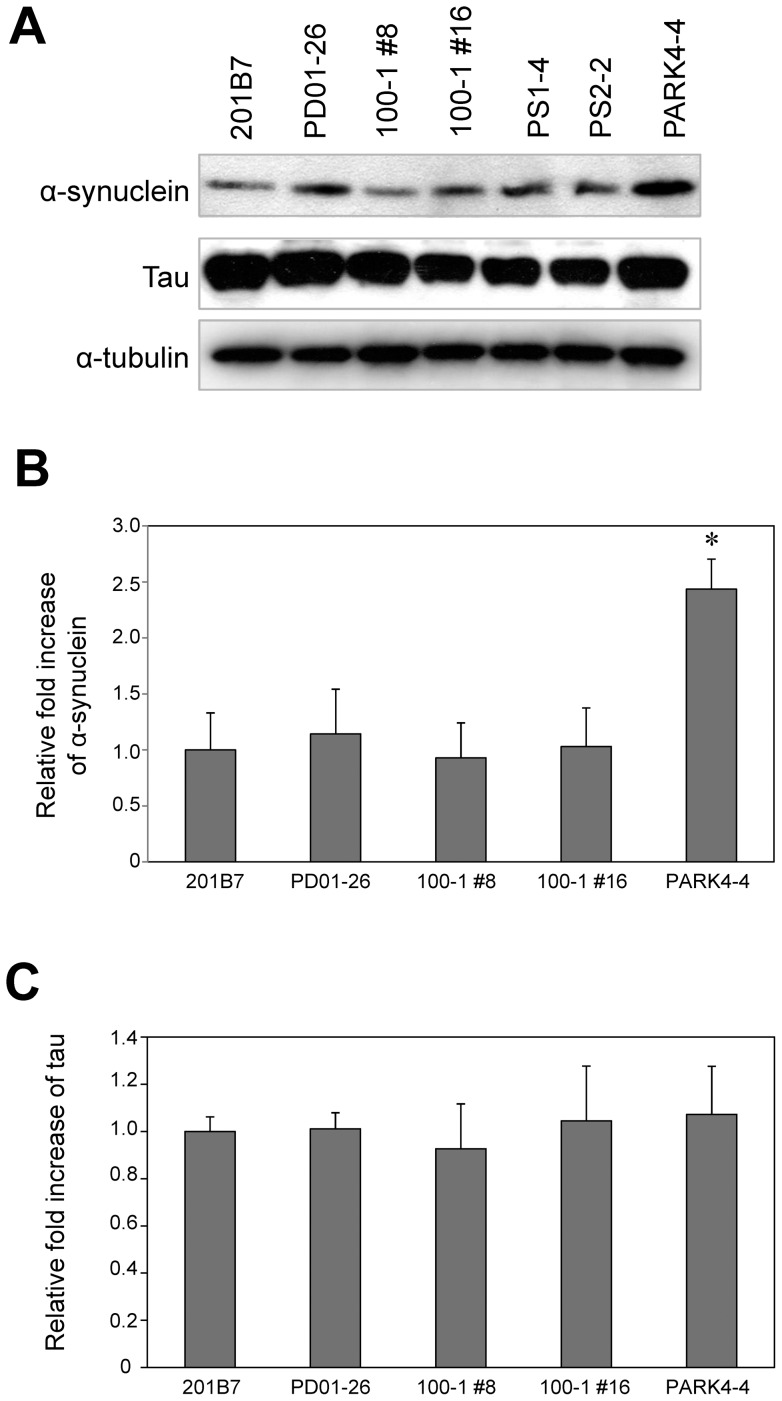
Expression of α-synuclein and tau by neurons differentiated from iPSCs 100–1 #8 and 100–1 #16, and the neurodegenerative disease-specific iPSCs. (A) Western blotting of α-synuclein and tau protein in the seven lines of neurons differentiated from iPSCs. Note that the level of α-synuclein expression by neurons differentiated from the PARK4-4 iPSCs is markedly increased. (B and C) Immunoblots were scanned and analyzed using densitometry. The level of α-synuclein (B) and tau (C) expression was normalized to the internal control α-tubulin. The histograms show expression relative to expression by 201B7. Data are from three independent experiments and are expressed as the mean ± standard deviation. Asterisks indicate a significant difference versus wild type as determined by one-way analysis of variance followed by Tukey-Kramer's post hoc test (*P* = 0.05).

Next, we assessed α-synuclein expression, which is another key pathological indicator of common neurodegenerative diseases. Parallel immunoblot analyses showed that, as expected, the level of α-synuclein in neurons differentiated from PARK4-iPSCs is high, due to triplication of the α-synuclein gene [4] (Figs. 6A and 6B). However, there were no differences with respect to the level of α-synuclein between neurons differentiated from centenarian-, FAD-, or sporadic PD-iPSCs. Furthermore, no significant differences in tau levels were detected among the neurons differentiated from these iPSCs (Figs. 6A and 6C). Collectively, these findings indicated that when used in conjunction with valid positive controls such as neurons differentiated from FAD- and PARK4-iPSCs, neurons differentiated from patient-specific iPSCs could be used in the study of β-amyloidopathy and α-synucleinopathy. In addition, our data suggest that iPSCs derived from centenarian donor cells could serve as a reliable super-control in studies of these late-onset neurodegenerative diseases.

## Discussion

The phenotypes of various hereditary neurodegenerative diseases can now be recapitulated in patient-specific iPSCs. In early studies, only congenital and pediatric disease phenotypes could be recapitulated, and phenotypes observed in iPSCs were reflected in abnormalities during early development. More recently, however, reports have appeared describing the recapitulation of phenotypes of late-onset neurodegenerative diseases, such as AD and PD, using patient-derived iPSCs [3]–[8], [14]. It is hoped that in the future, the use of patient-derived iPSCs can be expanded to include research into sporadic neurodegenerative diseases, which occur more frequently and impose a significant burden upon aging societies. Because biochemical and/or cellular abnormalities associated with sporadic neurodegenerative diseases (which are characterized by delayed onset of symptoms and slow progression) may not be prominent, comparing and analyzing subtle abnormalities linked to these disease processes must await the development of valid normative control iPSCs. Toward this end, we used a conventional method involving reprogramming with Oct3/4, Sox2, Klf1, and c-Myc to establish lines of iPSCs derived from centenarian donors who were extremely healthy, even at an advanced age. Our data indicate that our centenarian iPSCs and FAD- and PARK4-iPSCs are suitable for use in neurodegenerative disease research, especially with respect to β-amyloidopathy and α-synucleinopathy, and that these cells could serve as valid super-controls for use in studies of other late-onset diseases. Furthermore, it is expected that disease-specific phenotypes in patient-iPSCs would be diagnostic, and this property may lead to the development of novel diagnostic methods that utilize patient-iPSCs rather than invasive techniques such as brain biopsy. Our series of centenarian iPSCs and FAD- and PARK4-iPSCs would be useful as positive controls for both proteinopathies.

Several studies have reported that cellular senescence is a barrier to reprogramming due to upregulation of p53, p16INK4A, and p21CIP1 in aged cells [10], [15]–[19], suggesting that cells obtained from aging individuals may not be suitable for use in regenerative medicine research. Lapasset et al. recently demonstrated that generating iPSCs from senescent cells (cell cycle arrest) or from fibroblasts obtained from aged individuals (including centenarians) may require reprogramming with NANOG and LIN28a in combination with the four factors originally described by Yamanaka [10]. Fortunately, because the dermal fibroblasts we obtained from the 106- and 109-year-old centenarians grew well, we were able to establish centenarian-iPSCs using only the four factors without any difficulties. Although more efficient reprogramming techniques are now available, most of the disease-specific iPSCs that have been established to date were generated from dermal fibroblasts using Yamanaka's four original factors. The centenarian-derived iPSCs we established might therefore be more suitable to studies comparing disease-specific iPSCs from a biochemical or cell biological standpoint.

Both centenarian donors in this study showed cognitive dysfunction for approximately one year prior to death after their daily activities declined as a result of a leg fracture. However, both donors were extremely healthy, and did not suffer from dementia, movement disorders, or other serious diseases until an advanced age (Text S1). Although healthy aging is thought to result from complex interactions between genetic and environmental factors, it is assumed that from a genetic perspective two conditions are necessary and/or sufficient for exceptional longevity. First, an absence of disease risk factors is important for longevity. Healthy centenarians who have avoided common diseases are ideal controls for age-related disease studies [9]. Certainly, both of the centenarian donors in our study were negative for the APOE-ε4 variant (Text S1), which is the most significant established genetic risk factor for late-onset sporadic AD [20]–[23], and which is also associated with increased risk of early mortality [24], [25].

The second condition necessary and/or sufficient for exceptional longevity is the presence of factors that uniquely protect against common serious diseases [26]. Association studies have identified several genetic characteristics associated with longevity [27]–[29]. Centenarian-iPSCs could therefore serve as a novel material for studies aimed at uncovering key protective mechanisms that promote longevity. Although heat map analyses of the centenarian-iPSCs generated in this study showed that there were no significant differences compared to 201B7 iPSCs with respect to the expression of longevity related molecules (Figure S1), additional clones must be generated from cells obtained from other centenarians so that slight biochemical and/or cellular differences in iPSCs with large clonal variations can be characterized.

Geriatric welfare and medical care of persons suffering from late-onset neurological diseases, dementia, and Parkinson's disease are pressing issues in aging societies. Unfortunately, a number of clinical trials of disease modifying therapies or drugs to treat neurodegenerative diseases such as AD have failed. We hope that the results of our study will contribute significantly toward advancing understanding of late-onset neurodegenerative diseases such as AD and PD, and that our results will also contribute to establishing effective therapeutic strategies.

## Materials and Methods

### Cell culture and iPSC generation

After obtaining written informed consent from the families, fibroblasts were obtained postmortem from the abdominal wall of 106-year-old and 109-year-old female donors. PARK4-specific fibroblasts (ND27760) were obtained from the Coriell Cell Repository. Fibroblasts were cultured in Dulbecco's Modified Eagle's Medium (Gibco) containing 10% fetal bovine serum, 50 U/mL penicillin, 50 mg/mL streptomycin, and 1 mM L-glutamine. Methods for the maintenance of human dermal fibroblasts, lentiviral production, retroviral production, infection, stem cell culturing and characterization, RT-PCR, teratoma formation, *in vitro* differentiation, and neural induction were performed as described previously [6]. Generation of sporadic Parkinson's disease-derived iPSCs and FAD-iPSCs was carried out as described elsewhere [6]. Confocal images were acquired as described elsewhere [30]. All the experimental procedures used for skin-autopsy and biopsy and iPS production were approved by the Keio University School of Medicine Ethics Committee. If the donor lacks the capacity to consent for skin-autopsy and biopsy, proxy consent from the participant's families is authorized.

### Immunofluorescence staining of iPSCs and differentiated neurons

Immunofluorescence staining was performed using the following primary antibodies: anti-SSEA 3 (Abcam), anti-SSEA 4 (Abcam), anti-Tra-1–60 (Millipore), anti-Tra-1–81 (Millipore), anti-SSEA1 (Abcam), mouse anti-α-fetoprotein IgG (R&D Systems), anti-smooth muscle actin (Sigma), anti-βIII-tubulin (Chemicon), and anti- MAP-2 (Chemicon). DAPI (4, 6-diamidino-2-phenylindole; Molecular Probes) was used for nuclear staining. The secondary antibodies used were: anti-rat IgG, anti-mouse IgG, and IgM conjugated with Alexa Fluor 488 or Alexa Fluor 568 (Molecular Probes).

### Microarray analysis

Microarray analysis was performed as described in a previous report [6]. Briefly, human genome U133 Plus 2.0 GeneChip arrays (Affymetrix) were used for microarray hybridizations to examine global gene expression. Approximately 150 ng of RNA from each sample was labeled using GeneChip 3′IVT Express (Affymetrix), according to the manufacturer's instructions. All arrays were hybridized at 45°C for 16 h and scanned using an AFX GC3000 G7 scanner. Gene expression raw data were extracted using the AFX Gene Chip Operation System. Quality control was performed on the basis of Affymetrix quality control metrics. The data were analyzed using Gene Spring GX software, version 11.0 (Agilent). Microarray data can be accessed from the NCBI Gene Expression Omnibus (GEO) website under accession no. GSE28379 (The following link has been created to allow review of record GSE36667: http://www.ncbi.nlm.nih.gov/geo/query/acc.cgi?token=vnwvlecuqqwmelu&acc=GSE36667). The gene expression profiles for BJ fibroblasts (GSM248214) were downloaded from the GEO database.

### aCGH analysis

Microarray analysis was performed as described in a previous report [6]. Briefly, genomic DNA was labeled using the Enzo Genomic DNA Labeling kit. Hybridizations were performed on slides containing four arrays, with each array containing 622,060 *in situ* synthesized 60-mer oligonucleotides, representing 170,344 unique chromosomal locations (Agilent Technologies). Images of the arrays were acquired using a G2505CA microarray scanner (Agilent Technologies), and images were analyzed using Feature Extraction software, version 10.7 (Agilent Technologies). Oligonucleotides were mapped according to the human genome build NCBI 36. The data obtained were imported into Agilent Genomic Workbench using the aberration detection method 2 (ADM-2) algorithm (10.0 threshold) for further analysis.

### Telomere length analysis

Genomic DNA was extracted using DNeasy (Qiagen), according to the manufacturer's instructions. A total of 5 μg of DNA was digested with the restriction enzyme HinfI (TaKaRa, Japan), and the digested DNA was resolved on 0.8% agarose gels electrophoresed at 50 V for 5 h in 1X TAE buffer. Genomic DNA of HT1080, with a TRF length of slightly less than 5.0 kb [31], was use as a positive control for measurement of TRF length. Fractionated DNA fragments were transferred onto nylon membranes (Hybond-XL, GE Healthcare, USA) by an alkaline transfer technique using capillary blotting. Membranes were hybridized for 15 h at 42°C in 0.5 M Na_3_PO_4_ (pH 7.2) buffer containing 1 mM EDTA and 7% (w/v) SDS. A (CCCTAA)_4_ probe was labeled at the 5′ end with [γ-^32^P]ATP (Amersham, UK) using T4 polynucleotide kinase (TaKaRa, Japan). The membranes were washed in 40 mM Na_3_PO_4_ (pH 7.2) buffer containing 1 mM EDTA and 7% (w/v) SDS at 42°C for 2 h, after which they were dried with filter papers and exposed to Fuji Imaging Plates (Fuji Photo Film Co. Ltd., Japan) for 12 h at room temperature. Images of the blots were collected on a Typhoon 9400 image analyzer (Amersham) and analyzed using the ImageQuant program (Amersham).

### Quantitation of Aβ by ELISA

Differentiated neurons were cultured for 48 h, after which the conditioned medium was collected and analyzed using β-amyloid ELISA Kits (WAKO), according to the manufacturer's instructions.

### Immunoblot analysis

Cells were briefly sonicated in cold lysis buffer (50 mM Tris-HCl, pH 7.4, 150 mM NaCl, 0.5% NP-40, 0.5% sodium deoxycholate, 0.25% SDS, 5 mM EDTA, and protease inhibitor cocktail (Sigma)). The total concentration of protein in the supernatant was determined using a Bio-Rad protein assay kit. The proteins were separated using reducing SDS-PAGE on a 4–20% Tris-glycine gradient gel (Invitrogen) and then transferred to a polyvinylidene difluoride membrane (Millipore). The membrane was incubated with primary antibodies and then horseradish peroxidase-conjugated secondary antibodies. Detection was performed using enhanced chemiluminescence reagents as instructed by the supplier (PerkinElmer Life Sciences). The primary antibodies used in this study were: anti-α-synuclein BD (Transduction Laboratories), anti-tau (Thermo Scientific), and anti-alpha tubulin (Cell Signaling Technology). The relative fold-increase data shown in Figures 6B and 6C were determined by densitometry using an Epson ES-2000 scanner and Image J software (National Institutes of Health, Bethesda, MD).

## Supporting Information

Figure S1 ***In vitro*** **differentiation of iPSCs 100–1 #8, 100–1 #16, and 100–2 #1 via embryoid bodies.** Immunocytochemistry of βIII-tubulin (ectoderm), alpha smooth muscle actin (mesoderm), and α-fetoprotein (endoderm). Bar  = 50 μm.
(TIF)

Figure S2 **The transcriptome of 100–1 iPSCs.** (A) Hierarchical clustering of gene expression data for the indicated cell types. (B) Scatter-plot presentation of the expression values for all probe sets derived from genome-wide microarray expression data for the indicated cell types. (C) Expression profiles of longevity related molecules in 100–1 iPSCs shown in scatter plots against 201B7.
(TIF)

Table S1 **aCGH analysis of clones 100–1 #8 and 100–1 #16.**
(XLSX)

Text S1 **Clinical information about Donor 100–1 and 100–2.**
(DOCX)

## References

[1] Takahashi K, Tanabe K, Ohnuki M, Narita M, Ichisaka T (2007). Induction of pluripotent stem cells from adult human fibroblasts by defined factors.. Cell.

[2] Takahashi K, Yamanaka S (2006). Induction of pluripotent stem cells from mouse embryonic and adult fibroblast cultures by defined factors.. Cell.

[3] Nguyen HN, Byers B, Cord B, Shcheglovitov A, Byrne J (2011). LRRK2 mutant iPSC-derived DA neurons demonstrate increased susceptibility to oxidative stress.. Cell Stem Cell.

[4] Devine MJ, Ryten M, Vodicka P, Thomson AJ, Burdon T (2011). Parkinson's disease induced pluripotent stem cells with triplication of the alpha-synuclein locus.. Nat Commun.

[5] Seibler P, Graziotto J, Jeong H, Simunovic F, Klein C (2011). Mitochondrial Parkin recruitment is impaired in neurons derived from mutant PINK1 induced pluripotent stem cells.. J Neurosci.

[6] Yagi T, Ito D, Okada Y, Akamatsu W, Nihei Y (2011). Modeling familial Alzheimer's disease with induced pluripotent stem cells.. Hum Mol Genet.

[7] Israel MA, Yuan SH, Bardy C, Reyna SM, Mu Y (2012). Probing sporadic and familial Alzheimer's disease using induced pluripotent stem cells.. Nature.

[8] Jiang H, Ren Y, Yuen EY, Zhong P, Ghaedi M (2012). Parkin controls dopamine utilization in human midbrain dopaminergic neurons derived from induced pluripotent stem cells.. Nat Commun.

[9] Editorial NG (2011). Supersequencing the supercontrols.. Nat Genet.

[10] Lapasset L, Milhavet O, Prieur A, Besnard E, Babled A (2011). Rejuvenating senescent and centenarian human cells by reprogramming through the pluripotent state.. Genes Dev.

[11] Nori S, Okada Y, Yasuda A, Tsuji O, Takahashi Y (2011). Grafted human-induced pluripotent stem-cell-derived neurospheres promote motor functional recovery after spinal cord injury in mice.. Proc Natl Acad Sci U S A.

[12] Okada Y, Matsumoto A, Shimazaki T, Enoki R, Koizumi A (2008). Spatiotemporal recapitulation of central nervous system development by murine embryonic stem cell-derived neural stem/progenitor cells.. Stem Cells.

[13] Miura K, Okada Y, Aoi T, Okada A, Takahashi K (2009). Variation in the safety of induced pluripotent stem cell lines.. Nat Biotechnol.

[14] Koch P, Breuer P, Peitz M, Jungverdorben J, Kesavan J (2011). Excitation-induced ataxin-3 aggregation in neurons from patients with Machado-Joseph disease.. Nature.

[15] Li H, Collado M, Villasante A, Strati K, Ortega S (2009). The Ink4/Arf locus is a barrier for iPS cell reprogramming.. Nature.

[16] Kawamura T, Suzuki J, Wang YV, Menendez S, Morera LB (2009). Linking the p53 tumour suppressor pathway to somatic cell reprogramming.. Nature.

[17] Marion RM, Strati K, Li H, Tejera A, Schoeftner S (2009). Telomeres acquire embryonic stem cell characteristics in induced pluripotent stem cells.. Cell Stem Cell.

[18] Utikal J, Polo JM, Stadtfeld M, Maherali N, Kulalert W (2009). Immortalization eliminates a roadblock during cellular reprogramming into iPS cells.. Nature.

[19] Banito A, Rashid ST, Acosta JC, Li S, Pereira CF (2009). Senescence impairs successful reprogramming to pluripotent stem cells.. Genes Dev.

[20] Corder EH, Saunders AM, Strittmatter WJ, Schmechel DE, Gaskell PC (1993). Gene dose of apolipoprotein E type 4 allele and the risk of Alzheimer's disease in late onset families.. Science.

[21] Maestre G, Ottman R, Stern Y, Gurland B, Chun M (1995). Apolipoprotein E and Alzheimer's disease: ethnic variation in genotypic risks.. Ann Neurol.

[22] Myers RH, Schaefer EJ, Wilson PW, D'Agostino R, Ordovas JM (1996). Apolipoprotein E epsilon4 association with dementia in a population-based study: The Framingham study.. Neurology.

[23] Saunders AM, Schmader K, Breitner JC, Benson MD, Brown WT (1993). Apolipoprotein E epsilon 4 allele distributions in late-onset Alzheimer's disease and in other amyloid-forming diseases.. Lancet.

[24] Schachter F, Faure-Delanef L, Guenot F, Rouger H, Froguel P (1994). Genetic associations with human longevity at the APOE and ACE loci.. Nat Genet.

[25] Ewbank DC (2002). Mortality differences by APOE genotype estimated from demographic synthesis.. Genet Epidemiol.

[26] Sebastiani P, Solovieff N, Puca A, Hartley SW, Melista E (2010). Genetic signatures of exceptional longevity in humans.. Science 2010.

[27] Flachsbart F, Caliebe A, Kleindorp R, Blanche H, von Eller-Eberstein H (2009). Association of FOXO3A variation with human longevity confirmed in German centenarians.. Proc Natl Acad Sci U S A.

[28] Barzilai N, Atzmon G, Schechter C, Schaefer EJ, Cupples AL (2003). Unique lipoprotein phenotype and genotype associated with exceptional longevity.. JAMA.

[29] Sebastiani P, Montano M, Puca A, Solovieff N, Kojima T (2009). RNA editing genes associated with extreme old age in humans and with lifespan in C. elegans.. PLoS One.

[30] Ito D, Seki M, Tsunoda Y, Uchiyama H, Suzuki N (2010). Nuclear transport impairment of ALS-linked mutations in FUS/TLS.. Ann Neurol.

[31] Zhang DH, Zhou B, Huang Y, Xu LX, Zhou JQ (2006). The human Pif1 helicase, a potential Escherichia coli RecD homologue, inhibits telomerase activity.. Nucleic Acids Res.

