# Supplementary figures and images for: Establishment of Induced Pluripotent Stem Cells from Centenarians for Neurodegenerative Disease Research
Source: PLoS One. 2012 Jul 25;7(7):e41572. doi: 10.1371/journal.pone.0041572 (PMC3405135; doi:10.1371/journal.pone.0041572)

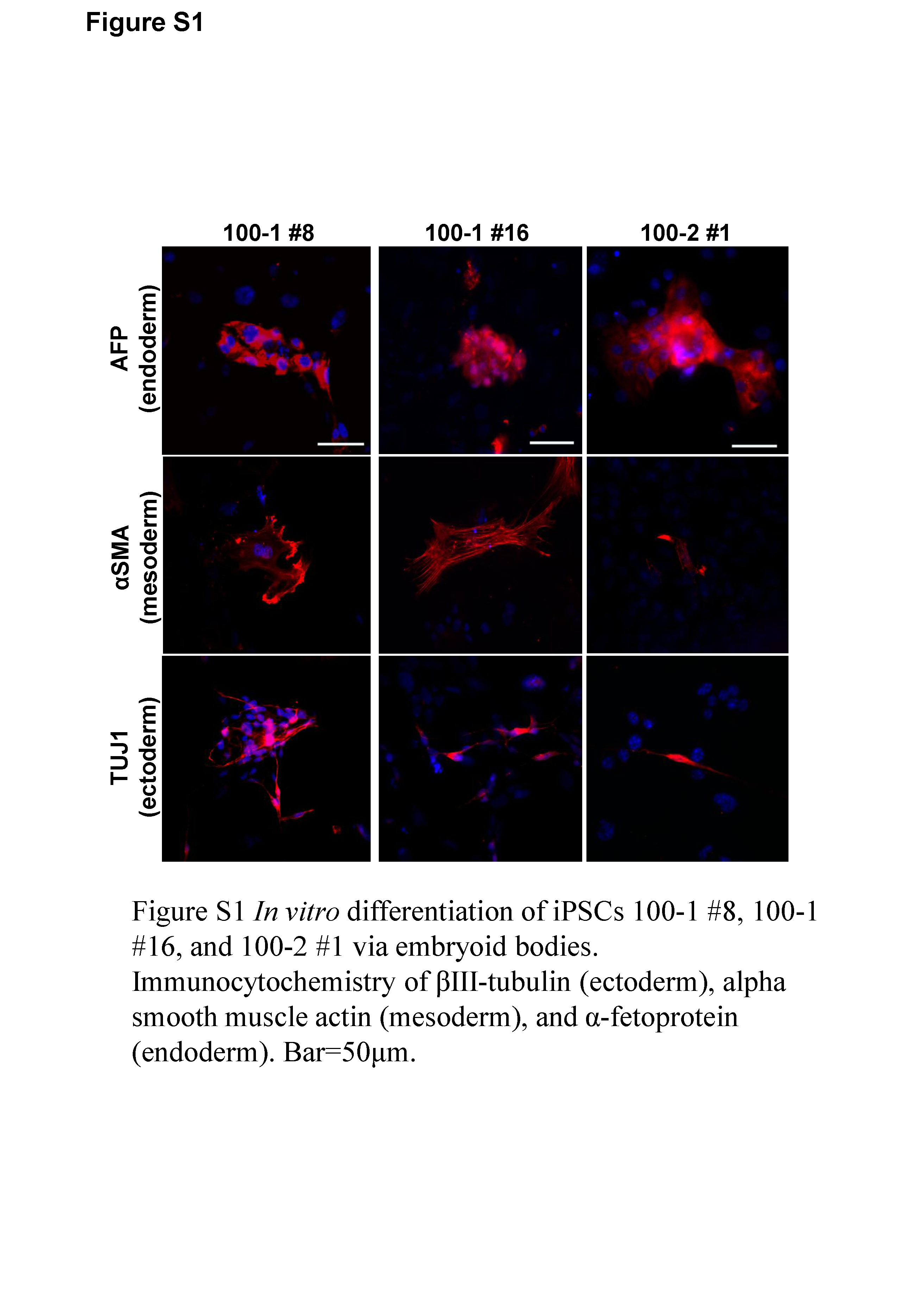

Supplement: Figure S1 — In vitro differentiation of iPSCs 100–1 #8, 100–1 #16, and 100–2 #1 via embryoid bodies. Immunocytochemistry of βIII-tubulin (ectoderm), alpha smooth muscle actin (mesoderm), and α-fetoprotein (endoderm). Bar = 50 μm. (TIF) [file pone.0041572.s001.tif]

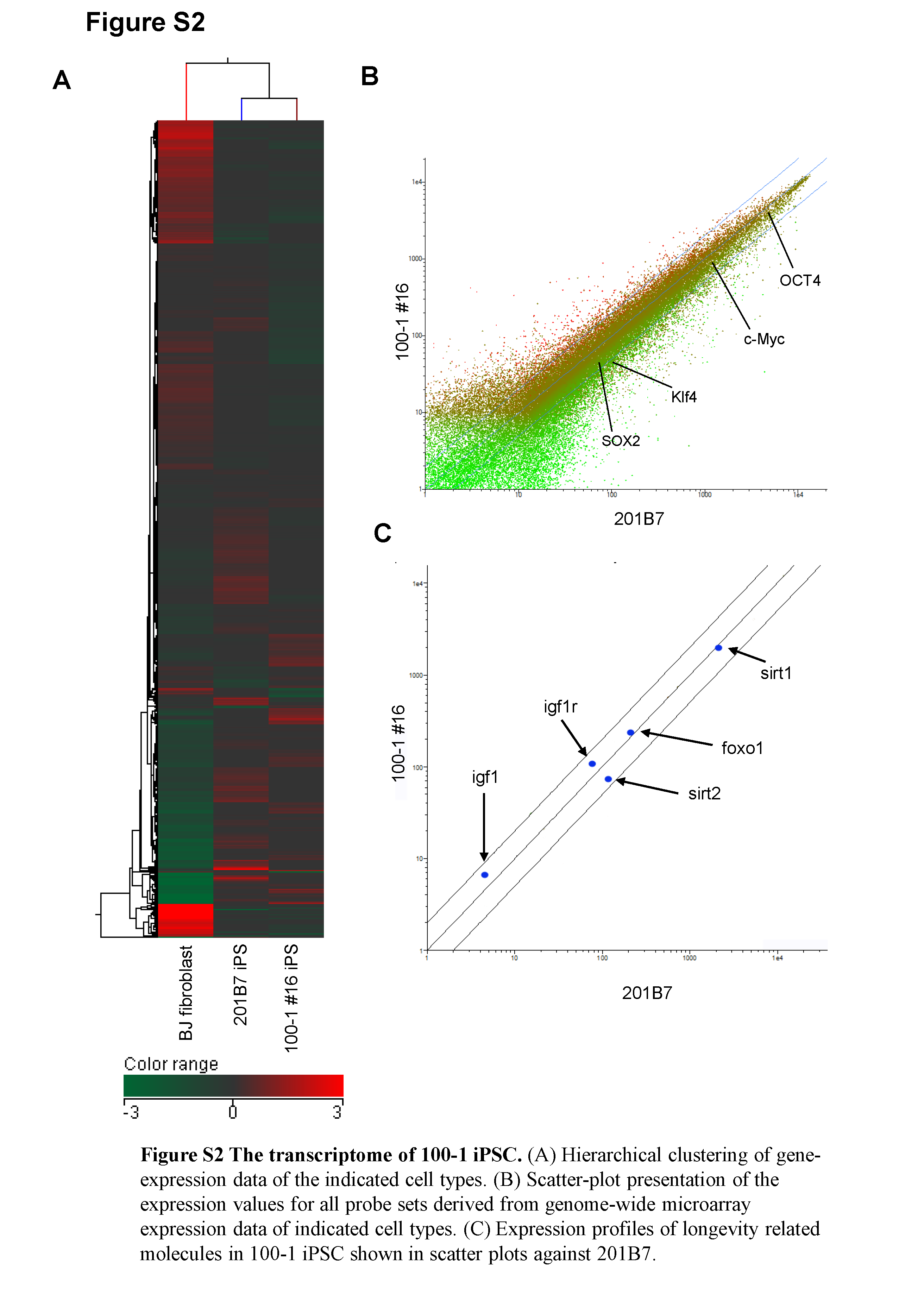

Supplement: Figure S2 — The transcriptome of 100–1 iPSCs. (A) Hierarchical clustering of gene expression data for the indicated cell types. (B) Scatter-plot presentation of the expression values for all probe sets derived from genome-wide microarray expression data for the indicated cell types. (C) Expression profiles of longevity related molecules in 100–1 iPSCs shown in scatter plots against 201B7. (TIF) [file pone.0041572.s002.tif]
