# Supplementary material for: Establishment of Induced Pluripotent Stem Cells from Centenarians for Neurodegenerative Disease Research
Source: PLoS One. 2012 Jul 25;7(7):e41572. doi: 10.1371/journal.pone.0041572 (PMC3405135; doi:10.1371/journal.pone.0041572)
Supplement: Text S1 — Clinical information about Donor 100–1 and 100–2. (DOCX) [file pone.0041572.s004.docx]

**Donor 100-1 (female)**

Medical history was taken from medical records and an interview with the donor’s son and granddaughter. The donor had been quite healthy and had not been treated for any chronic medical conditions until age 94. She received a license for a master of Japanese dancing at age 80. The donor was first hospitalized for cataract surgery at age 94 and was started on calcium blocker and nitrate treatment for hypertension at about the same age. At age 100 the donor’s Barthel Index was estimated at 90 points; she required some assistance with toilet use and use of stairs, but could do other activities alone. The donor’s Clinical Dementia Rating (CDR) at age 100 was estimated at 0.5. She demonstrated slight forgetfulness and slight difficulty with time relationships. The donor was able to walk until she sustained a fracture of the right metatarsal due to a fall at age 105. She was later admitted to the hospital with subfever and arthralgia. Following a diagnosis of polymyalgia rheumatica and urinary tract infection, steroids and antibiotics were administered for several weeks. The donor was left bedridden and required full assistance, and began to suffer a decline in cognitive function. The donor died due to aspiration pneumonia at age 106. Skin samples were obtained at 3 h postmortem after receiving informed consent from the family. The donor’s apolipoprotein E (APOE) genotype was ε3/3.

**Donor 100-2(female)**

Medical history was taken from an interview and an autobiographywith the donor’s daughters. The donor was quite healthy and had never been treated for any chronic medical conditions except for the fractures described below. She was first hospitalized for a right [femoral neck fracture](http://ejje.weblio.jp/content/femoral+neck+fracture) due to fall during her 70s, from which time she required the use of a crutch to walk. At age 100, her Barthel Index was estimated at 80 points, and she could walk with the assistance of a stick for more than 50 yards but was unable to use stairs and required assistance with bathing. Toilet use and dressing were independent, however. The donor’s CDR age 100 was estimated at 0.5. She demonstrated slight forgetfulness and slight difficulty with time relationships, but had no cataracts or deafness. After [fracturing her femur](http://ejje.weblio.jp/content/fracture+of+femur) and fibula at ages 107 and 108, respectively, the donor was wheelchair-dependent and her cognitive function began to gradually decline (Barthel Index = 20, CDR = 3). The donor died due to [senile decay](http://ejje.weblio.jp/content/senile+decay) at age 109. Skin samples were obtained at 3 h postmortem after receiving informed consent from the family. The donor’s APOE genotype was ε3/3.
